# Pherotype Polymorphism in *Streptococcus pneumoniae* Has No Obvious Effects on Population Structure and Recombination

**DOI:** 10.1093/gbe/evx188

**Published:** 2017-09-14

**Authors:** Eric L. Miller, Benjamin A. Evans, Omar E. Cornejo, Ian S. Roberts, Daniel E. Rozen

**Affiliations:** 1 School of Biological Sciences, Faculty of Biology, Medicine and Health, Manchester Academic Health Science Centre, University of Manchester, United Kingdom; 2 Institute of Biology, Leiden University, The Netherlands; 3 Norwich Medical School, University of East Anglia, Norwich, United Kingdom; 4 School of Biological Sciences, Washington State University

**Keywords:** competence, pneumococcus, quorum sensing, balanced polymorphism, horizontal gene transfer

## Abstract

Natural transformation in the Gram-positive pathogen *Streptococcus pneumoniae* occurs when cells become “competent,” a state that is induced in response to high extracellular concentrations of a secreted peptide signal called competence stimulating peptide (CSP) encoded by the *comC* locus. Two main CSP signal types (pherotypes) are known to dominate the pherotype diversity across strains. Using 4,089 fully sequenced pneumococcal genomes, we confirm that pneumococcal populations are highly genetically structured and that there is significant variation among diverged populations in pherotype frequencies; most carry only a single pherotype. Moreover, we find that the relative frequencies of the two dominant pherotypes significantly vary within a small range across geographical sites. It has been variously proposed that pherotypes either promote genetic exchange among cells expressing the same pherotype, or conversely that they promote recombination between strains bearing different pherotypes. We attempt to distinguish these hypotheses using a bioinformatics approach by estimating recombination frequencies within and between pherotypes across 4,089 full genomes. Despite underlying population structure, we observe extensive recombination between populations; additionally, we found significantly higher (although marginal) rates of genetic exchange between strains expressing different pherotypes than among isolates carrying the same pherotype. Our results indicate that pherotypes do not restrict, and may even slightly facilitate, recombination between strains; however, these marginal effects suggest the more likely possibility that the cause of CSP polymorphism lies outside of its effects on transformation. Our results suggest that the CSP balanced polymorphism does not causally underlie population differentiation. Therefore, when strains carrying different pherotypes encounter one another during cocolonization, genetic exchange can occur without restriction.

## Introduction

The Gram-positive pathogen *Streptococcus pneumoniae* is responsible for up to one million deaths annually (O’Brien etal. 2009). *S. pneumoniae* is naturally transformable, and this ability to take up and recombine extracellular DNA across a broad size range, from 15 bp to 19 kb (Mostowy etal. 2014), is associated with the acquisition of antibiotic resistance genes and with capsular switching (Croucher etal. 2014a, 2014b, 2011). Transformation in *S. pneumoniae* occurs following the quorum-dependent induction of “competence,” which is regulated by the secretion and detection of Competence Stimulating Peptide (CSP), encoded by *comC* (Pestova etal. 1996; Håvarstein etal. 2006). There are several alleles for *comC* and the gene encoding its cognate receptor, ComD, although the vast majority of isolates carry either of two dominant mature signals encoded by *comC*, that is, Csp-1 or Csp-2 (Pozzi etal. 1996; Whatmore etal. 1999; Evans and Rozen 2013). Although these different allele combinations, referred to as pherotypes, are known to be mutually unresponsive invitro, with isolates only responding to the CSP produced by isolates of their own pherotype (Iannelli etal. 2005), their role on the population structure of pneumococci remains unclear.

Two alternative hypotheses for the potential effects of multiple pherotypes on the population genetic structure of *S. pneumoniae* have been outlined. One hypothesis posits that because bacteria only bind and respond to their own CSP signal, pherotypes could ensure that bacteria only recombine with strains sharing the same pherotype, leading to a close association between pherotype and clonal structure (Håvarstein etal. 1997; Tortosa and Dubnau 1999). In the second hypothesis, CSP-activated fratricide, whereby CSP-induced cells use secreted bacteriocins to kill uninduced cells, could alternatively facilitate recombination between strains with varying pherotypes. Importantly, this hypothesis assumes that strains of one pherotype preferentially kill strains of other pherotypes, after which the induced strains recombine with the DNA liberated from lysed cells (Claverys etal. 2006; Claverys and Håvarstein 2007; Johnsborg etal. 2008; Cornejo etal. 2010). Although several recent studies have attempted to address the predictions of these models, results thus far are conflicting. Although results from Carrolo etal. (Carrolo etal. 2009, 2014) support the idea that pherotypes facilitate within-pherotype recombination and therefore underlie population differentiation, results from Cornejo etal. (2010) are more consistent with the alternative. Importantly, the results of these previous studies were limited to moderately small samples of strains, and analyses were performed on a limited number of markers (partial sequences of seven MLST loci), thereby reducing the ability to infer genomic-scale patterns of recombination. To overcome these limitations, our aim here is to address this question using a bioinformatics approach based upon analysis of recombination rates within and between pherotypes from 4,089 full pneumococcal genomes.

## Materials and Methods

### Genomic Data

We obtained 4,089 *S. pneumoniae* genomes from five publicly available sets (supplementary table S1, Supplementary Material online): 288 genomes from GenBank, which include 121 genomes of pathogenic strains from Atlanta, Georgia, the United States (Chancey etal. 2015); 3,017 genomes of carriage strains from Myanmar refugees in Maela, Thailand (Chewapreecha etal. 2014); 616 genomes of carriage strains from Massachusetts, the United States (Croucher etal. 2013b); 142 genomes of carriage strains from Rotterdam, the Netherlands (Bogaert etal. 2001; Miller etal. 2016); and 26 PMEN (Pneumococcal Molecular Epidemiology Network) genomes (McGee etal. 2001; Miller etal. 2016). These genomes were previously assembled and underwent quality control (Miller etal. 2016). We located *comC* and *comD* within these genomes using an iterative DNA reciprocal BLAST search as previously described (Miller etal. 2016). We excluded Rotterdam strain 724 and Maela strain 6983_6#45 from all pherotype analyses because the genomes of both strains carry two complete *comC* alleles encoding for both Csp-1 and Csp-2. We determined sequence types of genomes using MLSTcheck (supplementary table S1, Supplementary Material online; Page etal. 2016).

### Phylogenetic Analysis

To reconstruct the phylogenetic relationships of *comD*, we aligned full-length alleles using MUSCLE 3.8.425 (Edgar 2004) and Geneious 7.1.5 (Kearse etal. 2012). After deleting sites with 5% or more gaps, we inferred the GTR + I+ γ substitution model using jModelTest 2.1.7 (Darriba etal. 2012). We used MrBayes 3.2 (Huelsenbeck and Ronquist 2001; Ronquist and Huelsenbeck 2003) for phylogenetic reconstruction across four independent runs; we report clades with posterior probabilities ≥ 0.90 across these runs.

ComC in *S. pneumoniae* consists of a 41-amino acid peptide that is cleaved intracellularly into a 24-amino acid leader sequence and the secreted peptide signal called CSP. The short length of *comC* prevented us from reconstructing the phylogenetic history with confidence, as the mature CSP sequences were unable to be aligned. To indicate similarity between mature CSP signals, we used MUSCLE 3.8.425 (Edgar 2004) and Geneious 7.1.5 (Kearse etal. 2012) to align amino acid variants and produced a UPGMA phylogram of amino acid identity.

In order to characterize the overall genetic population structure in *S. pneumoniae*, we used data from a previously reported full genome phylogenetic tree (Miller etal. 2016) based on alignment to strain R6_uid57859 (Hoskins etal. 2001). Using a single reference strain restricted us to examining genetic diversity only in genes found in strain R6_uid57859; however the scale of computational analyses in this paper prevented us from repeating analyses with a range of diverse reference strains. In summary, we used genome sites found in at least 99.5% of all the above *S. pneumoniae* genomes (1,444,122 sites) and used the single best maximum likelihood tree [ln(likelihood) of − 3,2145,034.7] starting from 15 unique random trees and from 15 unique parsimonious trees, as calculated by RAxML v8.2.4 (Stamatakis 2006) and ExaML v3.0 (Kozlov etal. 2015). This tree included 82 genomes from pneumococcus Complex 3 (Croucher etal. 2013a) and 240 PMEN-1 genomes (Croucher etal. 2011); we excluded these strains from all of our further analyses because they were originally sampled based on membership to clonal complexes and are therefore biased with respect to population structure and pherotype. About 43 *Streptococcus sp.* viridans genomes were used as an outgroup for this tree, as previously reported (Miller etal. 2016).

For interspecific phylogenies, we identified *comC* and *comD* sequences from the available NCBI genomes for *S. infantis* (5 genomes), *S. mitis* (45 genomes), *S. oralis* (18 genomes), and *S. pseudopneumoniae* (40 genomes). The extensive sequence diversity in the mature CSP meant that we lacked confidence in aligning *comC*. Therefore, we used BAli-Phy v.2.3.6 (Suchard and Redelings 2006) for phylogenetic analysis because it estimates both the alignment and the tree simultaneously. We used the HKY+ γ model of substitution and constrained the leader sequence of *comC* to align together. BAli-Phy then estimated the full *comC* alignment and tree across eight independent runs using the S07 model of indel mutations.

Using MUSCLE 3.8.425 (Edgar 2004) and Geneious 7.1.5 (Kearse etal. 2012), we aligned the full-length comD sequences and deleted sites with more than 5% gaps. For further analysis, we used the filtered polymorphic sites from Gubbins (Croucher etal. 2015); any regions in individual sequences with evidence of recombination from Gubbins were replaced with N’s. We used a GTR + γ model of nucleotide substitution as determined by jModelTest 2.1.7 (Darriba etal. 2012) to reconstruct the phylogeny using MrBayes 3.2 across four independent runs.

### Estimating Population Structure

We used hierBAPS (Cheng etal. 2013) on the full genome (2,038,615 bp) alignment to strain R6_uid57859 (Hoskins etal. 2001) in order to examine population structure, whereas acknowledging that using a single reference strain reduces genetic variation to only those genes found in the reference strain. We used one run of 50, 60, 70, 80, 90, and 100 populations and four runs of 40 populations as upper bounds for the number of populations with between two and four levels of division. We found a constant number of 29–31 populations at the first level of division, and we used the aggregate of all runs at this level to divide genomes into populations for further analysis. We confirmed the inferred population structure using an orthogonal metric; for this, we measured nucleotide diversity across the entire genome by dividing the number of identical, nongapped sites by the total number of nongapped sites for pairwise combinations of genomes using Python 2.7.8. We then compared average nucleotide diversity between and within populations.

### Recombination Events

We utilized three separate approaches to assess whether there was more or less recombination within/between pherotypes than expected, based on the null expectation that recombination rates are proportional to the rates that strains encounter each other. The first two methods are based on pairwise tests between strains using GeneConv 1.81a (Sawyer 1999), whereas the third is based on results from Gubbins v2.1.0 (Croucher etal. 2015). Despite different limitations associated with each approach, it is reassuring that we found broad concordance in their associated output.

We used GeneConv 1.81a (Sawyer 1999) to detect recombination events of at least 100 bp between all pairwise comparisons of the 4,089 genomes. Briefly, this program detects continuous sections of DNA in pairwise alignments that have higher identity than the surrounding regions after accounting for monomorphic sites. We analyzed the genomes as “circular,” used Holm–Bonferroni correction for multiple testing (Holm 1979), and used a Gscale constant of 2, which scales to the number of mismatches allowed while detecting recombination events. The number of total recombination events detected by pherotype, by population, and by population/pherotype is reported in supplementary tables S2–S4 in Supplementary Material online, respectively.

We calculated the expected frequency of within- and between-pherotype recombination by assuming the null hypothesis that pherotype frequencies are indicative of how often pherotypes encounter each other; this encounter frequency is then an opportunity for genetic exchange that is proportional to actual recombination rates. Accordingly, if *i* represents the frequency of strains producing Csp-1, we expect two Csp-1 strains to encounter each other with frequency *i*^2^. To find the proportion of pairwise encounters that occur between strains carrying the same pherotype, we scaled the encounter frequency by the total encounter frequency of all strains, given that one must be a Csp-1 strain:

$$i^{2}+2i\left(1-i\right)$$

(encounter freq. of two Csp-1 strains) + (encounter freq. of a Csp-1 strain with a non-Csp-1 strain)

The proportion of pairwise encounters between strains expressing Csp-1 is then:

$$\frac{i^{2}}{i^{2}+2i\left(1-i\right)}$$

which reduces to:

$$\frac{i^{2}}{2i-i^{2}}.$$

We used this estimate to calculate the proportional expectation that recombination that occurs between strains carrying the Csp-1 pherotype. We compared this expected proportion with the observed proportion of within-Csp-1 strain recombination events, in which the number of detected pairwise recombination events between Csp-1 strains was divided by the total number of detected pairwise recombination events involving at least one Csp-1 strain. We repeated these calculations for each pherotype. The same approach was used to examine the expected frequency of between-pherotype recombination, except here the expected encounter rate between pherotypes is *2ij*, where *i* and *j* represent the frequencies of the focal pherotypes. As above, this leads to an expected proportion of between pherotype recombination as

$$\frac{2ij}{2i-i^{2}}$$

while focusing on pherotype *i* and as

$$\frac{2ij}{2j-j^{2}}$$

while focusing on pherotype *j*. This reflects that assumption that pherotypes *i* and *j* encounter other strains at rates proportional to their respective frequencies.

By dividing observed by expected recombination, we could assess if there was more or less recombination than expected by chance: values >1 indicate that there is more recombination than expected by chance, whereas values <1 indicate the opposite. We used the PropCIs package in R (R Core Team 2013) to estimate 95% confidence intervals for this ratio, and we tested if the observed recombination proportion differed significantly from the expected recombination proportion using Pearson’s χ^2^ statistic using R (R Core Team 2013). We additionally calculated observed and expected recombination proportions by classifying strains by populations in place of pherotype.

To examine within and between pherotype recombination in more detail, we next characterized the unique recombination events occurring between strains carrying the two dominant pherotypes (Csp-1 and Csp-2), which together comprise 95.5% of strains. By highlighting unique recombination events, the aim of this analysis was to remove the potentially biasing influence of vertical transmission, which could cause more ancient recombination events to be overrepresented compared with newer events. In order to identify unique recombination events, we grouped events that shared an identical start and end position in the full genome alignment. Next, we calculated the average proportion of these unique recombination events taking place between strains with Csp-1 and Csp-2 for each group (supplementary table S5, Supplementary Material online). We examined a range of cut-off points for the minimum proportion of strains that must be involved in each grouped recombination event, including: 0.05% of strains (≥2 strains, 1,663,312 events); 0.1% of strains (≥4 strains, 1,156,428 events); 0.5% of strains (≥20 strains, 381,084 events), 1.0% of strains (≥41 strains, 197,740 events), 2.5% of strains (≥102 strains, 59,980 events), and 5.0% of strains (≥204 strains, 19,428 events); this gradually focused the analysis on increasingly ancient recombination events. The expected proportion of between Csp-1 and Csp-2 recombination events was calculated as the expected encounter frequencies when only considering these two pherotypes, that is:

$$2*\;\frac{i}{\left(i+j\right)}*\frac{j}{\left(i+j\right)},$$

where *i* and *j* are the frequencies of Csp-1 and Csp-2, respectively.

To determine if our results were robust to the method chosen to detect recombination, we estimated within/between pherotype recombination using Gubbins v2.1.0 (Croucher etal. 2015) to estimate both the phylogeny and recombination break points for each of the 13 populations that contained more than one strain of both Csp-1 and Csp-2 pherotypes; we excluded populations that contained a single pherotype to minimize population-based recombination bias that would unduly influence a single pherotype. We first realigned each strain to the strain in each of the 13 populations with the highest N50 (supplementary table S6, Supplementary Material online) using Stampy v1.0.23 (Lunter and Goodson 2011). For the four populations aligned to complete, reference genomes, we inferred a single phylogeny per population. For each of the other nine populations, we inferred a phylogeny for each contig of the population’s reference genome. We only used Gubbins recombination events that were inferred to occur on the terminal branch leading to a strain’s sequence, so that only the strain within the population has the recombined sequence. This strain was designated as the recipient of the recombined sequence and we assumed that the strain had not changed pherotype since the recombination event. Gubbins can falsely label all but one strain as recipients of a recombination event in the case of an unrooted tree; we found no such cases in our analyses. To find the closest related strain to the recombination donor strain, we used BLASTn (Altschul etal. 1990; Camacho etal. 2009) on the full 4,089 strain genome database using the recombined sequence as the query to find the strains with highest bitscores. After removing the recombination recipient strain, we only considered comparisons in which all strains with the highest bitscore had the same pherotype, to best estimate the pherotype of the recombination donor strain. We used a binomial test using the frequency of the pherotype across all strains, and we used Holm–Bonferroni to correct for multiple testing (Holm 1979). To prevent diluting our power to find differences, we removed all population/pherotype combinations with less than ten comparisons (supplementary table S2, Supplementary Material online).

## Results

### *comC* and *comD* Diversity across *S. pneumoniae* Genomes and Geographic Sites

We estimated pherotype frequencies from 4,089 *S. pneumoniae* genomes that include extensive sampling in four geographic sites (table 1). The global population is dominated by two pherotypes (the mature signals encoded by the *comC* gene): Csp-1 (72.0% of strains) and Csp-2 (23.5% of strains). We also identified a novel variant in 2.1% of strains designated Csp-1_Short, which is identical to Csp-1 but with the final four residues deleted. Three other related variants contain between one and three “NFF” amino acid repeats, which we designate Csp-4_R1, Csp-4_R2, and Csp-4_R3, respectively. Note that Csp-4_R2 has previously been labelled Csp-4 while Csp-4_R3 is also called Csp-3 (Whatmore etal. 1999). About 0.27% of strains contained no *comC* sequence, which may result from incomplete genome sequencing; the low fraction of genomes without a *comC* sequence indicates strong selection against phenotypes that do not produce CSP. The overall frequency ratio of Csp-1::Csp-2 in previous studies was approximately 73.7%::26.3% after minority pherotypes were removed (Pozzi etal. 1996; Carrolo etal. 2009, 2014; Cornejo etal. 2010; Vestrheim etal. 2011); this is similar to the overall 75.4%::24.6% frequency ratio that we report after examining only Csp-1 and Csp-2 (table 1). However, we found significant differences in Csp-1::Csp-2 frequencies between the different strain collections (table 1), with Csp-1 frequencies ranging from 77.6% to 65.9%. The Maela strains, comprising 73.8% of the examined strains, are significantly different from the Massachusetts and Rotterdam strains (*P* = 2.14×10^−9^ and *P* = 1.46×10^−8^ respectively, two-sample proportion test) but not significantly different from the Atlanta strains (*P* = 0.527, two-sample proportion test). The Atlanta strains are significantly different from the Massachusetts strains (*P* = 0.0351, two-sample proportion test), with both of these sets not significantly different from the Rotterdam strains (*P* = 0.193 and *P* = 1.00 respectively, two-sample proportion test). Strain sets were broadly distributed throughout the phylogenetic tree (Miller etal. 2016).

**Table 1:** Frequencies of Pherotypes within Geographic Sites

		Frequency in Set				
Pherotype	Secreted Peptide	Maela, Thailand *N* = 3016	Atlanta, USA *N* = 121	Mass., USA *N* = 616	Rotterdam, The Netherlands *N* = 141	All 4,089 Genomes^a^
Csp-1	EMRLSKFFRDFILQRKK	0.736	0.702	0.643	0.634	0.720
Csp-1_Short	EMRLSKFFRDFIL	0.029	0	0	0	0.021
Csp-2	EMRISRIILDFLFLRKK	0.213	0.24	0.333	0.324	0.235
Csp-4_R1	EMRKMNEKSFNIFNFF—RRR	0.005	0	0.003	0	0.004
Csp-4_R2	EMRKMNEKSFNIFNFFNFF—RRR	0.004	0.008	0.018	0.035	0.007
Csp-4_R3	EMRKMNEKSFNIFNFFNFFNFFRRR	0.005	0	0.002	0	0.004
Other^b^	—	0.007	0.017	0.002	0.007	0.007
None	—	0.002	0.033	0	0	0.003

a This includes genome sets of variable geographic origin, such as the set of available genomes in GenBank.

b Other pherotypes each found in < 0.2% of genomes, as well as two genomes of both Csp-1 and Csp-2 pherotype.

**Fig. 1.**
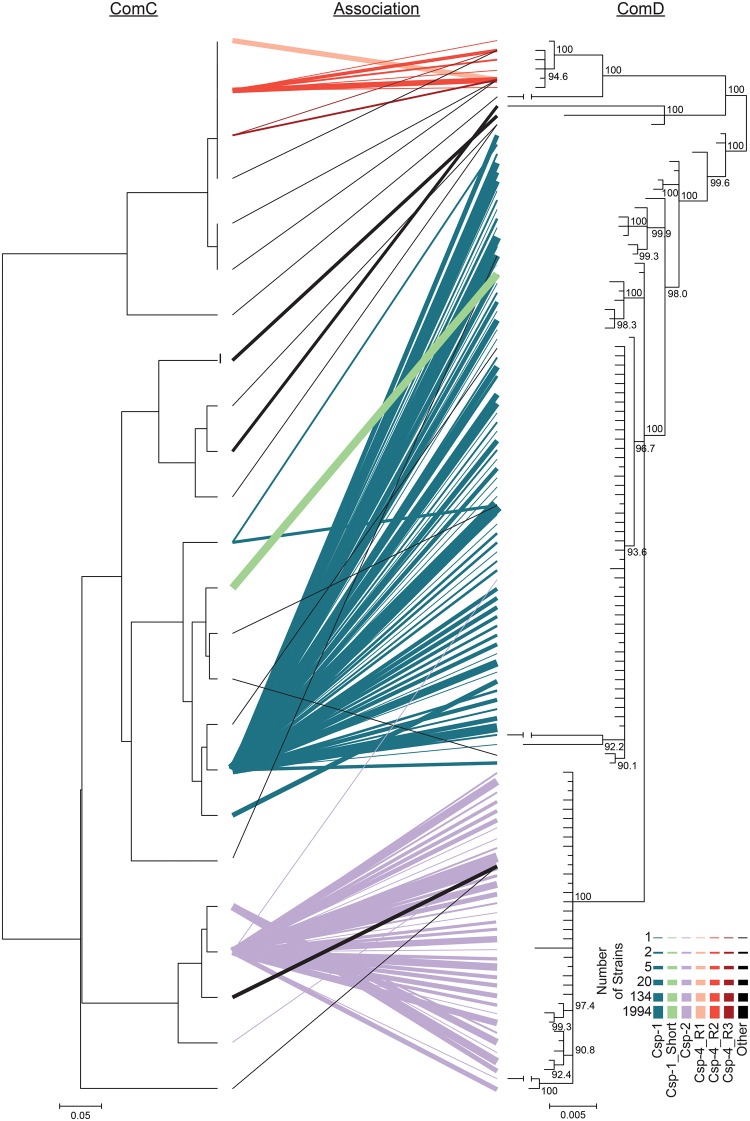
—Associations between ComC and *comD*. The UPGMA clustering of ComC genes is shown next to the inferred phylogenetic relationship between *comD* alleles. ComC amino acid variants (which encompass both the leader residues and the mature CSP) and *comD* nucleotide alleles found within the same genome are show as lines connecting the two phylograms, with thicker lines showing associations found in more strains. Line thickness is on a log scale. Classification of *comD* alleles is based on co-occurring CSPs within genomes, with a 99.8% correlation for the Csp-1 *comD* group, 99.2% correlation for the Csp-2 *comD* group, and 95.3% correlation for the Csp-4 *comD* group.

The polymorphism in CSP is mirrored in the histidine kinase receptor for CSP, *comD*. We found excellent concordance between the pherotype each strain carries and its corresponding *comD* sequence (fig. 1). About 935 of 936 strains with Csp-2 and full-length *comD* alleles contained *comD* alleles within a well-supported clade [posterior probability (PP) = 1.00] distinct from the other common (> 0.2% occurrence) pherotypes. Similarly, all *comD* alleles found with Csp-4 variants cluster in a well-supported (PP = 1.00) clade, with Csp-1 strains then forming a paraphyletic group with their *comD* alleles. The Csp-1 paraphyletic group also contains *comD* alleles associated with Csp-1_Short, which suggests this CSP may bind to the ComD receptor similarly to Csp-1. This supports tight functional linkage within three groups of pherotypes and ComD histidine kinases: Csp-1 (which includes Csp-1_Short), Csp-2, and the Csp-4 derivatives.

Interspecific gene trees of the signal gene *comC* (supplementary fig. S1, Supplementary Material online) lacked support for most interspecific clades. *comC* variants do not strictly cluster by species and Csp-4 derivatives form a clade with other nonpneumoniae *Streptococcus*, which could be indicative of: 1) horizontal transfer, which is not uncommon among related *Streptococcus* species (Balsalobre etal. 2003; King etal. 2005; Duesberg etal. 2008); or 2) the possibility that transspecific polymorphism is maintained in this locus as a balanced polymorphism (Ségurel etal. 2013; Gao etal. 2015). This second explanation is unlikely for *S. pneumoniae* given that 115 of 118 *S. pneumoniae* alleles form a well-supported clade that excludes other species in the interspecific *comD* phylogenetic tree of the histidine kinase receptor gene, in which we attempted to remove intragenic horizontal recombination (supplementary fig. S2, Supplementary Material online). By contrast, *S. mitis* and *S. pseudopneumoniae* freely intermix in all other clades, which is a pattern that supports transspecific polymorphism between these two species.

### Pherotype and Population Structure

**Fig. 2.**
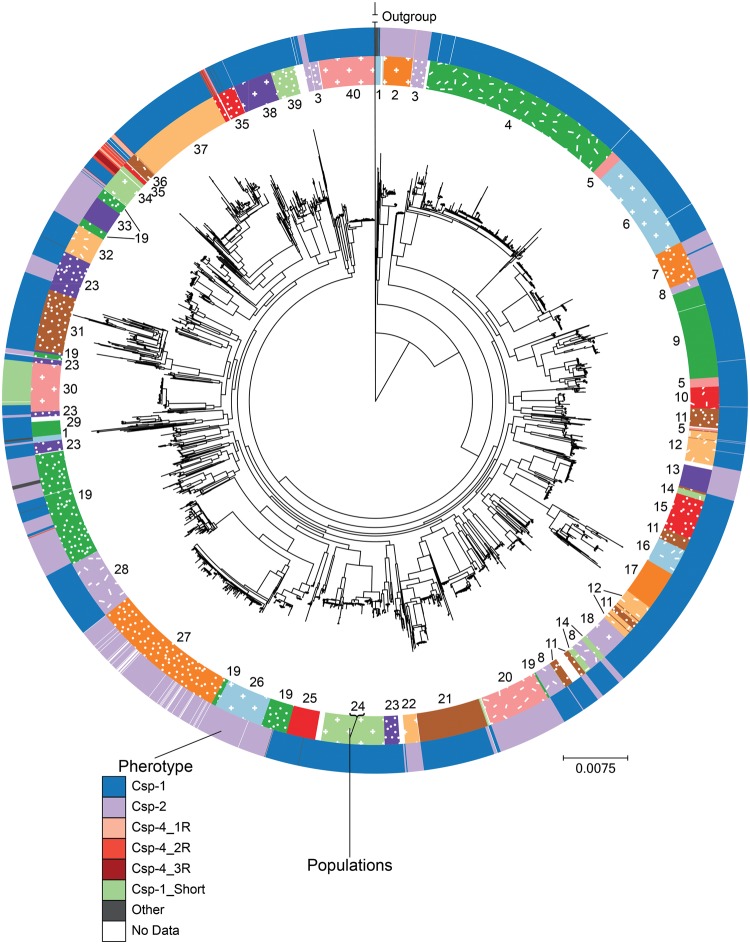
—Phylogenetic relationship between *Streptococcus pneumoniae* genomes. The inner coloured ring shows the population grouping of strains as determined by hierBAPS, shown as a number and a colour/pattern. Genomes not classified into a population are white in the inner ring. The outer ring denotes pherotype.

**Fig. 3.**
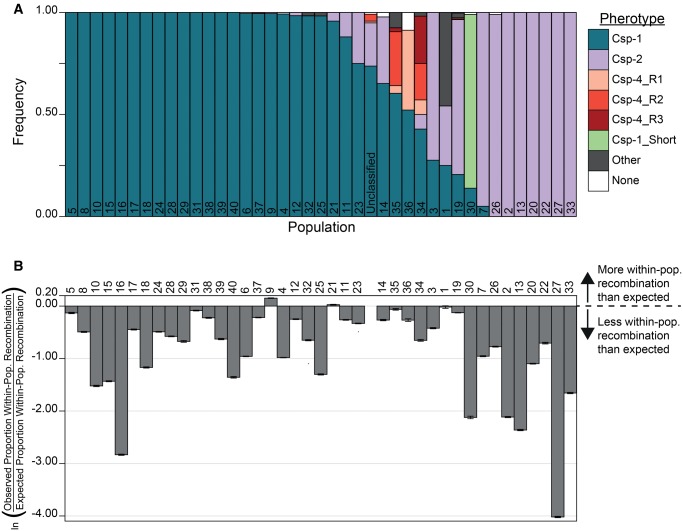
—Relationship between pherotypes and populations. (*A*) Distribution of pherotypes within each population. (*B*) Observed/expected fraction of within-population recombination for each population, with values <0 indicating less observed within-population recombination than expected. Error bars show 95% confidence intervals.

We inferred the population structure of these strains using 10 independent hierBAPS runs with a full-genome alignment (Cheng etal. 2013; fig. 2); 23 of the resultant populations were invariant across all runs. The remaining genomes were assigned to 1 of 17 populations that each contained 21 genomes or more (0.5% of the total number of genomes) that co-occurred in all ten runs. About 118 genomes (2.7%) could not be consistently classified into populations. Overall, this resulted in 40 estimated populations alongside the unclassified genomes. Ten (25%) of populations were not monophyletic on the full-genome phylogenetic tree, which is consistent with previous analysis of pneumococcal populations (fig. 2; Croucher etal. 2013b; Chewapreecha etal. 2014); this is due to the large amount of recombination in *S. pneumoniae* both obscuring deep branches and creating separate evolutionary histories for genes within the same genome. Consistent with the hierBAPS analysis, we estimated less genome nucleotide diversity within a population than between populations for 39 out of 40 populations (mean of per-within population nucleotide diversity = 0.00404; mean of per-between population nucleotide diversity = 0.0111; *P* < 1.2×10^−57^ except for population 1, *P* = 0.059; corrected Wilcox test). Pherotypes are not equally distributed across populations (fig. 3*A*), with 20 populations fixed for a single pherotype. Similarly, the site of collection affects population structure, as 16 of the 40 populations (representing 1,353 strains) consist solely of strains collected in Maela, Thailand (supplementary table S7, Supplementary Material online).

### Recombination Events within and between Pherotypes

To examine within versus between pherotype recombination, we first used GeneConv to compare the observed proportion of recombination events to the expected proportion of recombination events that assumes strains recombine randomly as a function of their expected encounter rates. Overall, we found no evidence that recombination is limited between populations. Although populations 9 and 21 had significantly more within-population recombination events than expected (fig. 3*B*), 37 other populations had significantly less within-population recombination events than expected (*P* ≤ 1.09×10^−6^ except for population 1, *P* = 0.395; Newcombe proportion test with Holm–Bonferroni correction). This is not an unexpected result, as within-population recombination events are harder to detect in more closely related genomes. The 30 monophyletic populations have a significantly lower ratio of observed/expected recombination events (*P* = 0.00575, Wilcoxon rank sum test) than the 10 polyphyletic populations (populations 1, 3, 5, 8, 11, 12, 14, 19, 23, and 35); this may reflect higher nucleotide diversity within the polyphyletic populations, which will again aid detecting within-population recombination events.

For analyses of recombination within and between pherotypes, we assume that strains do not change pherotype via recombination, a necessary assumption to avoid reconstructing all ancestral genomes to determine the correct pherotype at the time of historic recombination. To quantify these rates, and more generally to determine how often the pherotype itself is a substrate for recombination, we counted the number of total and unique recombination events that overlap with *comCD*. About 0.129% of detected recombination events with unique start/end positions (0.136% of all detected recombination events) overlap with this 0.0723% length of the genome (1,474 bp as aligned to R6). This region therefore has a higher rate of detectable recombination events, at 1.78 times as many (0.129%/0.0723%) recombination events compared with the genome average. Because recombination at this locus would only potentially lead to errors in scoring pherotypes for only 0.14% of detected recombination events, this potential source of error was ignored in subsequent analyses.

**Fig. 4.**
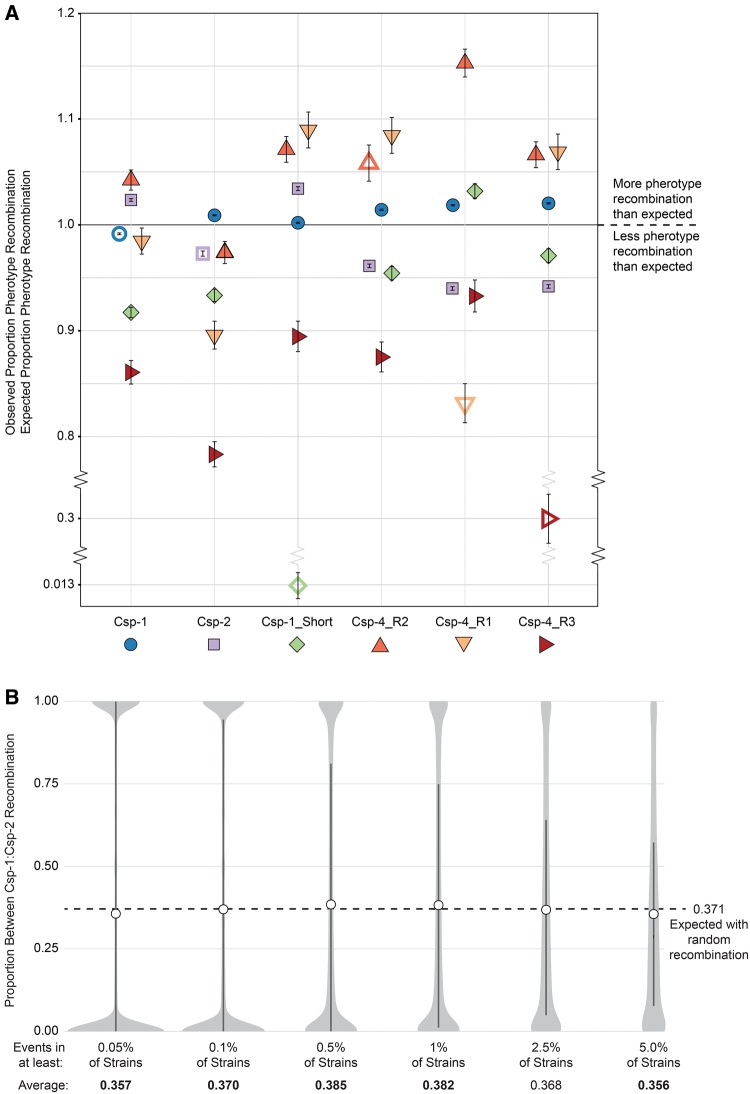
—Recombination between and within pherotypes. (*A*) Observed/expected fraction of recombination within and between pherotypes. Empty shapes are within-pherotype comparisons, whereas filled shapes show between-pherotype comparisons. Colours correspond to pherotypes as in figure 1. Error bars show 95% confidence intervals. (*B*) Distribution of the proportion of recombination events between Csp-1 and Csp-2, in which all recombination events with identical breaks points in the full-genome alignment are grouped as a single event. Light gray shapes show density estimate; dark bars incorporate the 25th to 75th percentile; white circles indicate the average proportion. Averages in bold are significantly different from the null expectation.

Figure 4 shows estimates of recombination for all common (> 0.2% of strains) pherotype combinations, with all observed recombination proportions differing significantly from those expected (*P* < 4.9×10^−6^; Newcombe proportion test with Holm–Bonferroni correction; fig. 4*A*). Of the 30 between-pherotype comparisons, 15 estimates of recombination proportions were significantly higher than expected, and 15 estimates were observed significant lower than expected, thus suggesting that there is no tendency for pherotype to either facilitate or diminish the chance of recombination. However, we found a higher observed recombination proportion than expected between strains expressing the two dominant pherotypes Csp-1 and Csp-2 (*P* < 10^−99^; Newcombe proportion test with Holm–Bonferroni correction), which together comprise nearly 95% of all strains. In total, recombination between these two pherotypes represents 34.7% of all recorded recombination events; thus an excess of recombination for these pairs may imply that the role of pherotypes is to marginally increase between-pherotype recombination. Consistent with this, five of the six within-pherotype observed recombination proportions are lower than expected, with Csp-4_R2 as the only exception (fig. 4*A*); three of these proportions are the lowest of all pherotype combinations within their respective pherotype.

A potential caveat of these results is that we consider every pairwise recombination event as independent. However, recombination events could have occurred at any time during the ancestry of these strains and then persisted via vertical descent, which may lead to biased estimates of recombination frequencies if a single, historic recombination event is counted in each of the multiple strains that descended from their common ancestor. As the true number of recombination events through history is unknown, we considered the opposite extreme, in which all pairwise strain recombination events with identical start and end positions in the full-genome alignment are considered a single recombination event. In figure 4*B*, we focused only on Csp-1 and Csp-2 pherotypes, and we considered unique recombination events found in a range of at least 0.05% of genomes (≥2 genomes) to at least 5.0% of genomes (≥204 genomes). This range of genomes allows us to examine the proportion of unique recombination events between Csp-1 and Csp-2 through time, as events found in at least 5.0% of strains will, on average, occur further in the past than events found in a minimum of 0.05% of strains. The mean proportion of between Csp-1::Csp-2 events of five out of six distributions are significantly different than a null expectation based on pherotype frequencies in the global population (*P* < 0.0253; Wilcox test for difference to μ = 0.371); however, no values differ by more than 4.0% from the null expectation of 0.371, with three averages significantly below and two averages significantly above the null value. This result indicates that any potential bias introduced by considering all recombination events independently is, at most, marginal.

As a final method to examine within and between pherotype recombination, we examined recent recombination events using an independent recombination detection method, Gubbins (Croucher etal. 2015). We used this method to identify and to place recombination events on an inferred, population-specific phylogeny of strains for the 13 populations containing at least two strains expressing either Csp-1 or Csp-2 in order to remove any population-associated effects. By focusing on recombination events that took place along the last branch before a strain’s genome, we were able examine recombination events that should have only one recipient strain. We then used BLASTn to find potential donor strains across all 4,089 strains with the highest bitscore. After removing the recombination recipient strain, we only considered comparisons in which all strains with the highest bitscore had the same pherotype, to best estimate the pherotype of the recombination donor strain. We analyzed the remaining 5,181 recombination events (table 2 and supplementary table S2, Supplementary Material online). After correcting for multiple comparisons, 2 of 12 Csp-1 comparisons and 4 of 9 Csp-2 comparisons have significantly higher proportions of intrapherotype recombination; 1 Csp-2 comparison has significantly lower amounts of intrapherotype recombination. Together, these results are consistent with those above: that pherotype has a marginal, if any, effect on recombination between pneumococcal strains.

**Table 2:** Reconstructed Pherotypes of Recipient and Donor Strains^a^

Recipient Pherotype	Population	Number of Comparisons	Frequency of Pherotype	Proportion of Comparisons with Intrapherotype Presumed Donor Strain	Uncorrected *P* Value	Corrected *P* Value
Csp-1	1	194	0.72	0.639	0.016	0.275
Csp-1	3	54	0.72	0.981	0	**1.90E-05**
Csp-1	7	10	0.72	0.7	1	1
Csp-1	11	175	0.72	0.794	0.029	0.428
Csp-1	12	26	0.72	0.615	0.274	1
Csp-1	14	199	0.72	0.678	0.206	1
Csp-1	19	411	0.72	0.725	0.869	1
Csp-1	21	465	0.72	0.8	0	**1.59E-03**
Csp-1	23	692	0.72	0.76	0.02	0.317
Csp-1	34	15	0.72	0.533	0.146	1
Csp-1	35	225	0.72	0.702	0.553	1
Csp-1	36	134	0.72	0.709	0.773	1
Csp-2	1	182	0.235	0.456	0	**1.58E-09**
Csp-2	3	175	0.235	0.091	0	**3.59E-05**
Csp-2	7	10	0.235	0.2	1	1
Csp-2	11	16	0.235	0.313	0.554	1
Csp-2	12	53	0.235	0.208	0.747	1
Csp-2	14	28	0.235	0.75	0	**2.99E-07**
Csp-2	19	1475	0.235	0.397	0	**4.49E-42**
Csp-2	21	12	0.235	0.417	0.168	1
Csp-2	23	195	0.235	0.369	0	**5.87E-04**
Csp-4_R1	35	19	0.004	0	1	1
Csp-4_R2	34	40	0.007	0.025	0.245	1
Csp-4_R2	35	209	0.007	0.048	0	**5.91E-05**
Csp-4_R3	34	73	0.004	0.055	0	**4.02E-03**
Csp-4_R3	35	94	0.004	0	1	1

Statistically significant corrected *P* values are in bold.

a Population/pherotype combinations with less than ten comparisons are found in supplementary table S2 in Supplementary Material online.

## Discussion

Several studies, including this one, have found that the two dominant pherotypes of the quorum-dependent regulator of competence in *S. pneumoniae* (i.e., Csp-1 and Csp-2) are maintained at relative frequencies of roughly 70:30 (Pozzi etal. 1996; Carrolo etal. 2009, 2014; Cornejo etal. 2010; Vestrheim etal. 2011). Our results also indicate that there is subtle geographic variation in the ratio of these dominant pherotypes, ranging from 66:34 to 78:22 (table 1), although the causes of these differences remain unknown. These results naturally lead to questions of how this pherotype ratio is maintained (to the near exclusion of other pherotypes), especially at similar levels in disparate geographic sites. The null explanation is that pherotypes are neutral with respect to bacterial fitness, although three lines of evidence counter this explanation. First, other studies suggest that comC is evolving under positive selection (Carrolo etal. 2009; Cornejo etal. 2010). Second, other *Streptococcus* viridans species have a higher diversity in CSP, ComC, and ComD (supplementary figs. S1 and S2, Supplementary Material online); this diversity is caused by a large number of pherotypes within each species as opposed to the two dominant pherotypes in *S. pneumoniae*. Third, each geographic site is comprised of strains from anywhere between 14 and 36 different populations (supplementary table S7, Supplementary Material online), yet each site maintains the approximate 70:30 ratio of major pherotypes. This interpopulation pattern between geographic sites is unlikely to occur through neutral mechanisms.

Irrespective of the factors maintaining pherotype polymorphism, what are the consequences of their maintenance for pneumococcal populations? Two divergent hypotheses have been explored to answer this question. One holds that these variants reinforce population subdivision by restricting recombination to strains that carry the same pherotype (Carrolo etal. 2009). The alternative hypothesis suggests that lysis of nonidentical pherotypes by the process of fratricide leads to interpherotype transformation and therefore the elimination of pherotype-specific population structuring (Cornejo etal. 2010). Our data, based on the analysis of recombination from more than 4,000 full genome sequences, do not clearly support the extreme version of either hypothesis. We find a very slight tendency toward increased between-pherotype recombination for the dominant pherotype classes (Csp-1 and Csp-2; fig. 4*A*), and these results clarify that pherotypes are not a barrier to recombination in this species. However, these data are not without limits, in particular the difficulty of inferring recombination among closely related strains, which could partly explain the lower frequencies of within-pherotype (fig. 4*A*) or within-population (fig. 3*B*) recombination. Because this should only have minimal influence on estimates of between-pherotype recombination frequencies, as strains carrying different pherotypes tend to come from different populations (fig. 3*B*), we do not anticipate that this bias will strongly influence our conclusions. In addition, using Gubbins, which is more sensitive for detecting recent recombination events, we found some evidence for an increased rate of intrapherotype recombination in some populations (2 of 12 for Csp-1 and 4 of 9 for Csp-2; table 2); however, there is no tendency for increased within-pherotype recombination in the majority of tested populations, and in one case the opposite result was obtained. Overall, these results, obtained via different approaches, seem to confirm that pherotype has a limited, if any, role on recombination frequencies between pneumococcal strains.

*S. pneumoniae* live in surface-associated biofilm communities, where in the case of single-strain colonization, they will be surrounded by clone-mates. If some of these cells become competent and lyse the others via competence-induced fratricide (i.e., induced production of bacteriocins that target uninduced cells), there will be little signature of this event at the genomic level. However, it is increasingly clear that multiple-strain infections are more common than previously thought; cocolonization within the nasopharynx is observed in up to 50% of individuals (Rodrigues etal. 2013; Wyllie etal. 2014), and because rare variants are less likely to be detected during sampling, this may be an underestimate of the true occurrence of cocolonization. In these cases, there is opportunity for genetic exchange between different genotypes. If the coinfecting genotypes express the same pherotype, both will respond to the same peptide signal and both genotypes could, in principle, release DNA that will be available to the other. However, if the genotypes express different pherotypes, then the genotype that first initiates competence may lyse the other nonresponding genotype via competence-induced fratricide, leading to more unidirectional uptake. Although recombination during single pherotype infections could reinforce any pre-existing association between pherotype and population structure, recombination during mixed-pherotype infections would cause this association to decline or disappear. Our results are most consistent with this latter scenario.

How often do mixed pherotype colonization events occur? If the likelihood of colonization is random with respect to pherotype, then the probability that both pherotypes will be found to co-occur is simply twice the product of these relative frequencies. In two recent studies where this has been measured, mixed pherotype infections are common in cases where multiple strains are seen (47.5% and 57.1% in strains from Portugal and Norway, respectively) and do not vary from the null expectation of random colonization (Vestrheim etal. 2011; Valente etal. 2012). These results have several important implications. First, they suggest that fratricide during the induction to competence has minimal, if any, impact on the within-host competitive dynamics of co-occurring strains. Second, they suggest that opportunities for recombination between strains expressing different pherotypes are widespread. Accordingly, we would predict pherotypes to have little direct influence on population structure, a prediction borne out by the results presented here and elsewhere (fig. 3*A*; Cornejo etal. 2010). This, of course, does not preclude structure arising from other influences, for example, serotype specific immunity, differences in antibiotic resistance or other attributes leading to biases in colonization. However, pherotypes neither appear to underlie this structure nor to eliminate it due to ubiquitous interpherotype recombination (fig. 4*A*).

A final explanation for a fixed pherotype ratio is that diversity is maintained for reasons wholly distinct from their effects on transformation. In addition to the capacity to become transformed, CSP induces more than 150 pneumococcal genes, and only a small fraction of these are required for DNA uptake and recombination (Peterson etal. 2000, 2004). Different concentrations of CSP are required for competence activation between Csp-1 and Csp-2 with their respective receptors (Iannelli etal. 2005; Carrolo etal. 2014), which determine the population density at which CSP induces these genes and could drive large phenotypic differences between pherotype based only on *comC* and *comD* variation. We were unable to find genomic loci that coassociates with CSP via selection using a genome-wide association study with pherotype as a phenotype (unpublished results), which suggests that any ecological differentiation between phenotype may be caused only by differences in *comC* and *comD*. However, Carrolo etal. (2014) find striking differences in the ability for strains expressing Csp-1 and Csp-2 to form biofilms, with Csp-1 strains producing biofilms with greater biomass. Although the authors suggest that this difference may lead to differences in the colonization success and transmissibility of isolates, epidemiological data discussed above are not consistent with this possibility; cocolonization with multiple pherotypes occurs as often as expected by chance (Vestrheim etal. 2011; Valente etal. 2012). An alternative, which could lead to a form of balancing or frequency-dependent selection maintaining pherotypes at intermediate frequencies, is the possibility that differences in biofilm-associated biomass that influence stable colonization may trade-off with reductions in transmissibility. By this scenario, Csp-1-expressing strains may form more robust biofilms during colonization, whereas strains expressing Csp-2 are more proficient at dispersal. Although testing these possibilities is beyond the scope of this work, these ideas can potentially be examined empirically in both invivo or invitro models. Furthermore, they make the prediction that carriage duration should vary as a function of pherotype, a possibility that could potentially be retrospectively examined from epidemiological studies.

Although our results are unable to clarify the evolutionary factors that lead to the origin and maintenance of pherotypes in *S. pneumoniae*, our comprehensive analyses using different approaches demonstrates the long-term effects of this polymorphism on recombination in this species. In summary, while pherotypes can weakly facilitate recombination between the major pherotype classes (and only in some populations), this effect is very marginal and has no evident impact on population structure of this pathogen. Explanations for pneumococcal population structure therefore lie outside of pherotypes, and indeed, explanations for pherotypes may lie outside of their effects on recombination.

## Supplementary Material

Supplementary data are available at *Genome Biology and Evolution* online.

## Supplementary Material

Supplementary Data
